# Self-assessment of the mental health status in older adults in Poland: a cross-sectional study

**DOI:** 10.1186/s12888-017-1557-y

**Published:** 2017-12-01

**Authors:** Mateusz Cybulski, Lukasz Cybulski, Elzbieta Krajewska-Kulak, Urszula Cwalina

**Affiliations:** 1 Department of Integrated Medical Care, Faculty of Health Sciences, Medical University of Bialystok, 7a M. Sklodowskiej-Curie str., 15-096 Bialystok, Poland; 2 National Security student, Faculty of Social Sciences, University of Warmia and Mazury in Olsztyn, Olsztyn, Poland; 3 Department of Integrated Medical Care, Faculty of Health Sciences, Medical University of Bialystok, Bialystok, Poland; 4 Department of Statistics and Medical Informatics, Faculty of Health Sciences, Medical University of Bialystok, Bialystok, Poland

**Keywords:** Beck depression inventory (BDI), Depression, GHQ-28, Older adults, Elderly, Health status, Mental health

## Abstract

**Background:**

Demographic aging of society poses numerous challenges, including the provision of health care to the elderly population. According to World Health Organization data, the most frequent mental disorders in the senior population are: dementia, depression, and drug and alcohol addiction. The aim of this study was to subjectively assess mental health status (the severity of non-psychotic symptoms of mental functions and depressive symptoms) in older adults of Bialystok (Poland).

**Methods:**

The study included 300 people – inhabitants of Bialystok and its surrounding areas – aged over 60: 100 residents of a nursing home, 100 senior students of the University of the Third Age in Bialystok, and 100 senior students of the University of a Healthy Senior. Two standardized psychometric scales were used in the study: the General Health Questionnaire (GHQ-28) and the Beck Depression Inventory (BDI).

**Results:**

The median GHQ total point value equaled 26 points, which indicated possible non-psychotic mental disorders. The overall BDI score showed that respondents had a subjective feeling of depressive symptom intensification at the level of 11 points out of 63 points, which indicated minor depressive disorders. Positive and statistically significant correlations were observed between suspicion of non-psychotic mental disorders and the occurrence of depressive symptoms both without distribution into groups and with distribution into sex, group affiliation, and age.

**Conclusions:**

Subjective assessment of mental health status in older adults, inhabitants of Bialystok, was negative. Social and demographic characteristics (sex, group affiliation, age) analyzed in the study, played no significant role in the assessment of depressive and non-psychotic mental symptom occurrence. Residents of the nursing home were characterized negatively in terms of subjective assessment mental health status from the other two study groups.

## Background

The aging of a population poses a significant challenge to public health, both in social and health terms. By 2020, more than one million Poles will be 90 years old, and by 2035 more than 25% of Poles will be 65 years old and older. In 2060, Poland will have one of the oldest populations in Europe [1].

At the beginning of the twenty-first century, about 20% of people aged over 55 suffered from mental disorders in the USA [2]. Over a few subsequent years, global statistics indicated that this problem affected seniors in most countries of the world [3]. According to World Health Organization data, the most common mental disorders in the older adult population include: dementia, depression, and addiction to alcohol and drugs – particularly benzodiazepine and opioids [4].

Therefore, it was interesting to investigate depressive and non-psychotic mental symptoms among the elderly from different groups in Bialystok, Poland, and to compare the data we obtained with data from other countries. No similar studies have ever been conducted in Poland. Approximately 15% of adults aged 60 and over, in the world suffer from some form of mental disorder, hence the importance of this study. It is advisable to conduct such studies among older adults in Poland in order to obtain data that will allow to compare the mental health situation in Poland and other countries.

The aim of this study was to subjectively assess the mental health status (the severity of non-psychotic symptoms of mental functions and depressive symptoms) of older adults of Bialystok (Poland), and to compare the data we obtained with data from different regions of Poland and other countries. Our hypothesis was that the older adults will assess their mental health status negatively, and that the nursing home residents – compared with all study groups – would be characterized by the worst severity of non-psychotic symptoms of mental functions and the presence of depressive symptoms. Due to the high prevalence of mental disorders in older adults in the world, we also consider it to be an important health and social problem in the study groups.

## Methods

### Participants

The study was carried out in three groups. Group I consisted of senior students of the University of a Healthy Senior (100 people), held at the Faculty of Health Sciences of the Medical University of Bialystok. The participants of group II were senior students of the University of the Third Age in Bialystok (100 people). Group III consisted of residents of the Nursing Home in Bialystok (100 people).

The study included 300 people aged over 60 – inhabitants of Bialystok and the surrounding areas. The biggest groups consisted of people aged 60 to 69 (51.3%; *n* = 154), 34% of the respondents were 70 to 79 (*n* = 102) and 14.7% 80 and over (*n* = 44). The arithmetic mean of the respondents’ age was 70.8 years, and standard deviation was 7.99. The study included 213 (71%) women and 87 (29%) men.

Another criterion for inclusion in the study, besides age and place of residence, was the absence of identified dementia or mental retardation in the potential respondents. Their presence were excluded on the basis of the conducted clock drawing test and analysis of medical documentation. Each participant had to give written consent to participate in the study and could withdraw from it at any stage.

The test group size depended on the number of University students and residents of the nursing home. For the purpose of the study, the authors collected 100 complete surveys in each subgroup. There were 193 people living in a nursing home, including people under the age of 60, as well as people with mental retardation or dementia, who did not meet the criteria. Those people were excluded from the study based on a diagnosis confirmed by a specialist physician. More copies of the research tools were distributed, but not all of them were returned to the authors of the study. We distributed 150 copies of questionnaires among the students of the University of a Healthy Senior and residents of the nursing home, and 200 copies among the participants of the University of the Third Age. The number of returned questionnaires was 127 in group I, 143 in group II, and 111 in group III, but not all returned surveys were complete. After analyzing all the returned questionnaires authors received 100 complete questionnaires form each group.

### Measurements and procedure

The study used two standardized psychometric scales: the General Health Questionnaire (GHQ-28) by Goldberg (1979), adapted by Makowska and Merecz [5]; and the Beck Depression Inventory (BDI) by Beck (1961), adapted by Parnowski and Jernajczyk [6].

GHQ is a screening instrument used for the assessment of mental health in adults in the general population [7]. It enables estimating the severity of non-psychotic mental symptoms, and identifying people who may very likely develop this type of disorder [8, 9]. There are several versions of the questionnaire – the basic, long version consisting of 60 items (GHQ-60), and shorter versions – developed by exclusion of some questions. The GHQ-28 version was developed as a result of the GHQ-60 factor analysis. Thanks to this scale, we can obtain data about somatic symptoms, anxiety, depression, insomnia, and social functioning disorders in addition to information about the general mental health state [10]. Respondents should answer the questions independently, assessing their life situation and mental state using a 4-level scale. The results are calculated using Likert’s method. Individual answers are given values from 0 to 3 [11]. This tool allows for the overall assessment of current mental health status, as well as diagnosis of specific symptoms, using the questionnaire’s four subscales: somatic symptoms – questions 1–7 (GHQ-28-A); anxiety and insomnia symptoms – questions 8–14 (GHQ-28-B); impaired daily functioning – questions 15–21 (GHQ-28-C); and depression symptoms – questions 22–28 (GHQ-28-D) [12]. There is no actual relationship between the results of the subsections. The maximum amount of points that can be obtained is 84. Higher values indicate more positive results. Using this method, a result of 23/24 points is the basis for suspecting non-psychotic mental symptoms [13]. Cronbach’s alpha coefficient of the analyzed scale is 0.9–0.95 [14].

There are three main advantages of this diagnostic tool. First, the GHQ-28 is shorter, requiring approximately 3 to 5 min to fill in the whole questionnaire. Additionally, it can be applied in primary-care conditions, where most of the minor psychiatric disorders arise. Furthermore, apart from providing an overall assessment, the GHQ-28 contains four scales that provide additional information [15]. However, there are some limitations to GHQ-28 use. The GHQ is likely to detect transient disorders which are likely to remit after minimal treatment. Indeed, most of the “false positives” are minor disorders of this sort. Similarly, it is likely to miss disorders of a very long duration, if respondents have come to accept their symptoms as “normal” for them. However, it is simple to detect such cases, either from their medical records or by adding a few more questions [16]. Additionally, the questionnaire is not available free of charge and must be purchased.

The BDI scale is a diagnostic screening tool used for measuring the intensity of depressive symptoms. It contains 21 phrases with assigned subscales and answers from 0 (lack of symptoms) to 3 points (the most severe symptoms) [17]. A result of 21 or more points suggests the occurrence of depressive symptoms. For people with diagnosed depression, a result of 0 to 9 points indicates the least severe symptoms of depression, 10 to 16 points - mild depression, 17 to 29 points - moderate depression, and 30 to 63 points - severe depression [18]. For the original version, Cronbach’s alpha coefficients are 0.27 to 0.74 in the control group and 0.39 to 0.70 in the group with depressive disorders. For the whole scale, Cronbach’s alpha coefficient is 0.93 and 0.92, respectively [17].

Advantages of the BDI include its ease of use, applicability to a diverse study group, and the fact that it has been the subject of numerous studies since its creation. Some studies has shown that the BDI is able to consistently and precisely estimate current levels severity of depression in many various conditions [19]. Because the BDI is self-reported questionnaire, there is a risk that respondents may exaggerate their answers. Furthermore, the BDI can only be used to measure the occurrence of depression perceived by a patient. It is not a typical diagnostic tool and has to be used in connection with other scales in order to provide proper analysis of the respondents’ current mental status.

The respondents from I and II group completed the questionnaires independently after receiving detailed explanation of the research procedure from members of the research team. Additional explanations and instructions were also included with every questionnaire. In group inhabitants of the nursing home (III), the respondents were interviewed directly by psychologists and occupational therapists employed by that institution.

### Procedure and ethical considerations

The study was performed from February to June 2016. The research conforms with the Good Clinical Practice guidelines, and the followed procedures were in accordance with the Helsinki Declaration. The research was approved by the Bioethics Committee of the Medical University of Bialystok (statute no. R-I-002/365/2015).

### Statistical analysis

The data were processed using Microsoft Excel 2013 and statistically analyzed using Statistica Data Miner C QC PL. Pearson’s Chi-square (χ^2^) test was used to analyze the dependence of qualitative features. Shapiro-Wilk’s test was used to assess the normality of distribution of quantitative features. Normal distribution of quantitative features was not found, therefore, the features were analyzed using non-parametric methods. U Mann-Whitney’s test was used to compare two groups, and the ANOVA Kruskal-Wallis’ test with post-hoc tests to compare three groups. Additionally, Spearman’s rank correlation coefficient was used. Study results of *p* < 0.05 were regarded as statistically significant.

## Results

### Mean GHQ-28 and BDI scores with regard to sex, group affiliation, and age

Table 1 shows mean GHQ-28 and BDI scores with regard to sex, group affiliation, and age. The median GHQ total point value was 26 points. The overall BDI results showed that the respondents had subjective feelings of increased severity of depressive symptoms at a level of 11 of 63 possible points. No differences were found between women and men in regard to any of the abovementioned variables. Taking into consideration the mean values of the analyzed scales in terms of group affiliation, nursing home (NH) residents had the highest results, while the students of the University of a Healthy Senior (UHS) the lowest. Statistically significant differences were also found between the respondents from UHS and NH in terms of BDI. Statistical analysis showed that results in the group of NH residents were statistically significant regarding: subjective assessment of somatic symptoms (GHQ-28-A), subjective assessment of social dysfunctions (GHQ-28-C), subjective assessment of depressive symptoms (GHQ-28-D), and subjective feeling of the severity of depressive symptoms (BDI). The BDI results of NH residents were also statistically significant. We analyzed the results of the particular scales in terms of respondent age. People aged 60–69 were the most numerous group (more than 50%). The youngest respondent was 60 years old, while the oldest 98. Statistically significant differences in relation to the age groups were found in case of subjective assessment of social dysfunctions (GHQ-28-C) between respondents aged 60–69 and those respondents aged 80 and up; and subjective feeling of the severity of depressive symptoms (BDI) between the age groups mentioned above as well as between respondents aged 70–79 and individuals over the age of 80. In each of the mentioned cases, the 80 and over group obtained significantly greater values compared with younger respondents.

**Table 1:** Mean GHQ-28 and BDI scores with regard to sex, group affiliation, and age

	Women *N* = 213		Men *N* = 87		*p*	UHS (I) *N* = 100		UTA (II) N = 100		NH (III) N = 100		*p*		60–69 years old (I) N = 154		70–79 years old (II) *N* = 102		80 years old and more (III) *N* = 44		*p*		Total *N* = 300	
	$\overline{x}\pm Sd$	Me	$\overline{x}\pm Sd$	Me		$\overline{x}\pm Sd$	Me	$\overline{x}\pm Sd$	Me	$\overline{x}\pm Sd$	Me			$\overline{x}\pm Sd$	Me	$\overline{x}\pm Sd$	Me	$\overline{x}\pm Sd$	Me			$\overline{x}\pm Sd$	Me
GHQ-28-A	8.92 ± 3.45	9.0	9.08 ± 3.75	9.0	0.813	8.27 ± 3.44	8.0	8.83 ± 3.24	9.0	9.79 ± 3.76	9.0	I-II I-III II-III	0.958 0.019 0.249	8.82 ± 3.36	8.0	8.76 ± 3.52	9.0	9.91 ± 4.05	10.0	I-II I-III II-III	1.00 0.283 0.378	8.96 ± 3.53	9.0
GHQ-28-B	7.5 ± 4.38	7.0	6.91 ± 4.96	6.0	0.133	6.69 ± 4.21	6.0	7.14 ± 4.23	6.5	8.15 ± 5.08	7.5	I-II I-III II-III	1.00 0.133 0.652	7.41 ± 4.13	7.0	7.12 ± 5.06	6.5	7.52 ± 4.82	6.5	I-II I-III II-III	0.845 1.00 1.00	7.33 ± 4.55	7.0
GHQ-28-C	8.79 ± 3.57	7.0	8.83 ± 4.16	7.0	0.625	7.64 ± 2.89	7.0	8.53 ± 2.85	7.0	10.24 ± 4.72	9.0	I-II I-III II-III	0.099 < 0.001 0.133	8.11 ± 3.16	7.0	8.93 ± 3.66	7.0	10.93 ± 4.9	10.0	I-II I-III II-III	0.413 0.005 0.152	8.8 ± 3.74	7.0
GHQ-28-D	3.3 ± 2.65	2.0	3.23 ± 3.04	2.0	0.346	2.58 ± 2.17	2.0	3.19 ± 2.37	2.0	4.07 ± 3.42	3.0	I-II I-III II-III	0.140 < 0.001 0.304	3.26 ± 2.59	2.0	2.96 ± 2.64	2.0	4.09 ± 3.48	3.0	I-II I-III II-III	0.401 0.619 0.072	3.28 ± 2.77	2.0
GHQ-28_total	28.51 ± 10.9	27.0	28.05 ± 13.34	24.0	0.163	25.18 ± 9.63	23.0	27.69 ± 9.57	27.0	32.25 ± 14.1	29.0	I-II I-III II-III	0.170 < 0.001 0.206	27.6 ± 9.95	25.5	27.77 ± 12.16	25.0	32.45 ± 14.91	29.5	I-II I-III II-III	1.00 0.393 0.269	28.37 ± 11.64	26.0
BDI	12.84 ± 8.23	11.0	11.34 ± 8.33	8.0	0.085	10.28 ± 6.65	8.5	10.87 ± 7.86	9.0	16.07 ± 8.96	14.5	I-II I-III II-III	1.00 < 0.001 < 0.001	11.2 ± 7.34	11.0	12.53 ± 8.57	10.0	16.34 ± 9.5	14.0	I-II I-III II-III	0.929 0.003 0.05	12.41 ± 8.27	11.0

*Abbreviations: GHQ-28-A* General Health Questionnaire (somatic symptoms), *GHQ-28-B* General Health Questionnaire (symptoms of anxiety and insomnia), *GHQ-28-C* General Health Questionnaire (social dysfunctions), *GHQ-28-D* General Health Questionnaire (symptoms of depression), *GHQ-28* General Health Questionnaire, *BDI* Beck Depression Inventory, *SD* Standard deviation, *Me* Median, *NS*0 Not significant, *UHS* University of the Healthy Senior; *UTA* University of the Third Age, *NH* Nursing Home

### Subjective assessment of the presence of depressive symptoms in respondents according to BDI and suspicion of non-psychotic mental symptoms in respondents according to GHQ-28 with regard to sex, group affiliation, and age

Table 2 shows subjective assessment of the presence of depressive symptoms in respondents according to the BDI and suspicion of non-psychotic mental symptoms in respondents according to the GHQ-28 with regard to sex, group affiliation and age. A statistically significant relationship was found between group affiliation and the presence of depressive symptoms. The lowest percentage of the presence of depressive symptoms was observed in the UHS student group, while the highest among NH residents. A statistically significant relationship was found between the occurrence of depressive symptoms and age. The lowest percentage of the presence of depressive symptoms was observed in the group of people aged 60–69, while the highest among the oldest respondents. After analyzing the occurrence of suspected non-psychotic mental symptoms using the GHQ-28, a statistically significant relationship was found between group affiliation and the results presented in the study. Particular attention should be paid to differences between the results in the UHS student group (more than half of the respondents had 23 points and more) and the NH resident group (nearly ¾ of the respondents had at least 23 points).

**Table 2:** Assessment of the presence of depressive symptoms in respondents according to BDI and suspicion of non-psychotic mental symptoms in respondents according to GHQ-28 with regard to sex, group affiliation, and age

		BDI scores		*p*	GHQ-28 scores		*p*
		Below 21 points *N* = 245	21 and more points *N* = 55		Below 23 points *N* = 245	23 and more points *N* = 55	
Women *N* = 213	n	173	40	0.755	72	141	0.073
	%	81.22	18.78		33.80	66.20	
Men *N* = 87	n	72	15		39	48	
	%	82.76	17.24		44.83	55.17	
UHS *N* = 100	n	90	10	<0.001	46	54	0.044
	%	90.00	10.00		46.00	54.00	
UTA *N* = 100	n	89	11		36	64	
	%	89.00	11.00		36.00	64.00	
NH *N* = 100	n	66	34		29	71	
	%	66.00	34.00		29.00	71.00	
60–69 *N* = 154	n	134	20	0.025	60	94	0.521
	%	87.01	12.99		38.96	61.04	
70–79 *N* = 102	n	80	22		38	64	
	%	78.43	21.57		37.25	62.75	
80 and more *N* = 44	n	31	13		13	31	
	%	70.45	29.55		29.55	70.45	

*Abbreviations*: *BDI* Beck Depression Inventory, *GHQ-28* General Health Questionnaire, *NS* Not significant, *UHS* University of the Healthy Senior, *UTA* University of the Third Age, *NH* Nursing Home

### Relationship between the raw results of particular scales, taking into consideration social and demographic characteristics

**Fig. 1:**
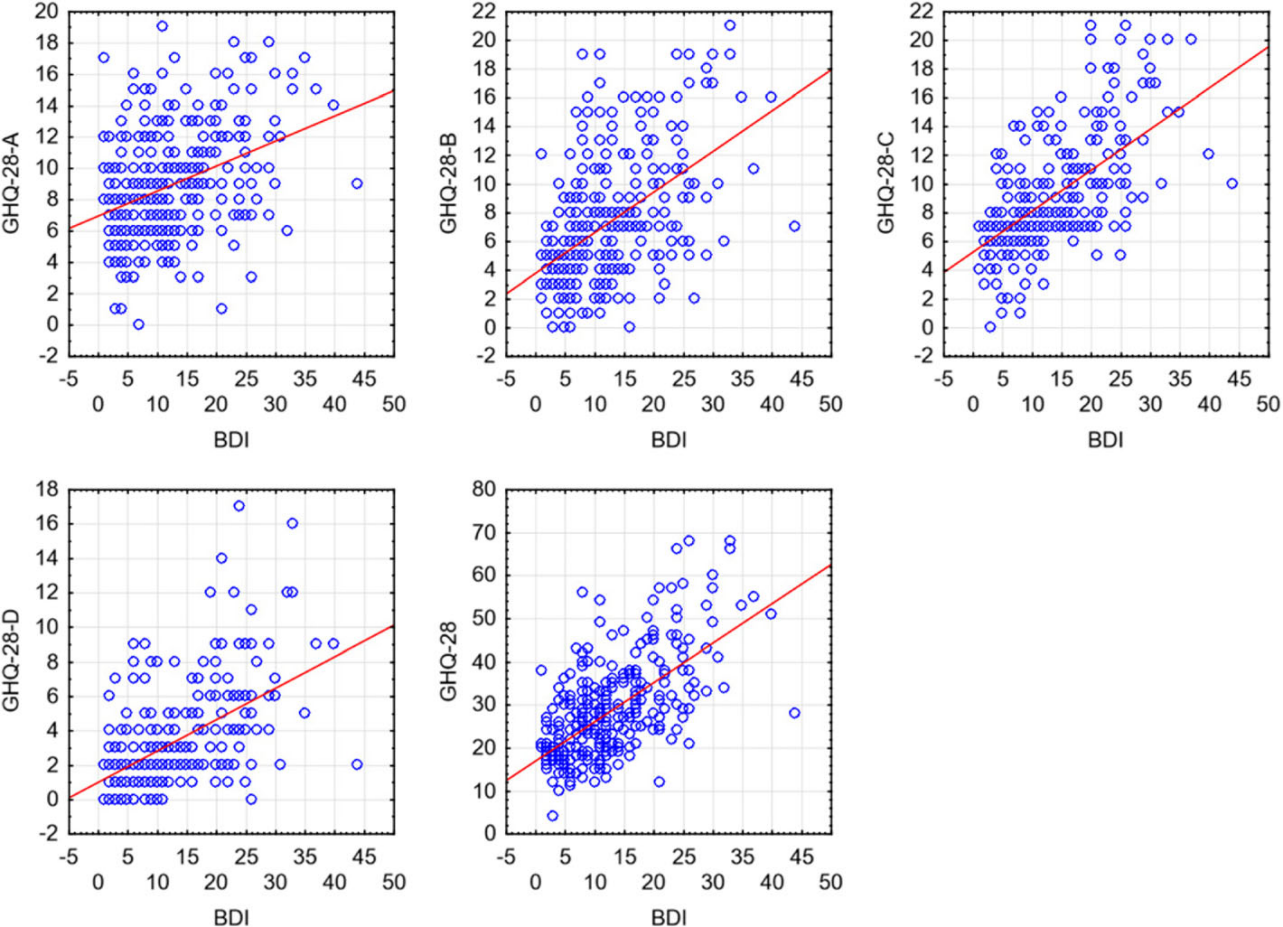
Scatter diagrams of the overall result and the results of individual subscales of the GHQ-28, and the results of the BDI. Abbreviations: GHQ-28-A, General Health Questionnaire (somatic symptoms); GHQ-28-B, General Health Questionnaire (symptoms of anxiety and insomnia); GHQ-28-C, General Health Questionnaire (social dysfunctions); GHQ-28-D, General Health Questionnaire (symptoms of depression); GHQ-28, General Health Questionnaire; BDI, Beck Depression Inventory

We also decided to analyze the relationship between the raw results of particular scales, taking into consideration social and demographic characteristics according to which the respondents were analyzed. No statistically significant relationship was found between the results of the BDI and the GHQ-28 and respondents’ age. However, positive and statistically significant relationships were reported between the suspicion of non-psychotic mental symptoms (GHQ-28) (*r* = 0.627, *p* < 0.001) with regard to particular subscales (GHQ-28-A – *r* = 0.365; *p* < 0.001; GHQ-28-B – *r* = 0.511, *p* < 0.001; GHQ-28-C – *r* = 0.603, *p* < 0.001; GHQ-28-D – *r* = 0.505, *p* < 0.001), and the occurrence of depressive symptoms (BDI) both without distribution into groups and with distribution by sex, group affiliation, and age. Figure 1 shows scatter diagrams of the overall result, the results of particular subscales of GHQ-28, and the BDI results.

## Discussion

### Suspicion of the presence of non-psychotic symptoms

The main non-psychotic mental disorders in the elderly are anxiety disorders [20]. These disorders reduce function and quality of life and may increase risk for other diseases such as depression, dementia, and cardiovascular disease [21]. The prevalence of anxiety disorders in older adults varies according to studies. In the article by Mackenzie et al., in the large NESARC study that included over 12,000 adults aged 55 and older, the frequency of these disorders was 2.8% [22]. Similarly, the article by Grenier et al., in a large epidemiologic study of older adults in Quebec, found that the combined incidence of syndromal and subthreshold anxiety was 26.2% [23]. In the study by Kirmizioglu et al. [24], prevalence for all types of anxiety disorders was 17.1%.

There are relatively few data on the prevalence of personality disorders in the elderly people. The aging advances might be expected to decline the frequency of personality disorders such as aggression, impulsivity, etc. However, co-occurring mental problems can increase their severity, for example older people are often socially withdrawn due to depressive disorders [25]. The incidence of personality disorders among older adults has been estimated to be about 10% [26–28]. The frequency of one or more personality disorders in the elderly in late-life are from 3 to 13% [29]. In addition, personality disorders were related with disability as well as somatic and mental disorders [30].

In our study, the median of total GHQ point value indicated a suspicion of the presence of non-psychotic mental symptoms, but the prevalence of non-psychotic symptoms was slightly lower than previously reported in other studies. Overall GHQ-28 result was higher for women than for men. A similar relationship and results were found in the study involving the Dutch population [31] and in the study by Apidechkul [32]. The results of the studies carried out by Kilic et al. [33] showed higher GHQ results for women than for men.

Zare et al. [34] showed a significant difference between the mean overall result and the mean results of GHQ-28 subscales among respondents. In that study, older adults were characterized by worse health status than other respondents [34]. In the study by Momeni and Karimi [35], the overall GHQ result and the results of the subscale of social dysfunction were almost identical to the results in our study, but there were certain differences in comparison with the other subscales. A medical history of chronic diseases in older adults was correlated with symptoms of anxiety and depression reported in other studies [36, 37]. The occurrence of advanced depression ranged from 1% to 16% in older adults in private homes or institutions [38], which is close to the results reported in our study.

The data indicated a higher prevalence of depressive symptoms in nursing homes residents, or even clinically relevant depression, which may also be related to age. Thus, the results confirmed our hypothesis that the studied disorders occur most frequently in residents of nursing homes. Nordtug et al. [39] carried out an interesting study, showing that mean GHQ-28 scale results were lower for patients with chronic obstructive pulmonary disease than for patients with dementia. Krzych et al. [40] presented similar results in a study conducted on cardio-surgical patients with an average age of 71. Makowska and Merecz [41] reported even better results in an assessment of mental state in their study. The GHQ questionnaire also showed mental and social disorders in patients with backache in the study of Iranian women [42] and in the Greek studies carried out in primary health care facilities [43]. Our results, compared with the results of other authors, confirmed the hypothesis that mental disorders are an important health and social problem in the studied group of seniors.

In our study, women had higher mean results in terms of the abovementioned features, however, the difference was not meaningful or statistically significant. In the study by Datta et al. [44], a model of multiple linear regression showed no significant relationship between the respondents’ sex and the total GHQ result and the results of the subscales of somatic symptoms, social dysfunctions, and advanced depression. However, women had significantly higher results for anxiety and sleeping disorders [44]. The predominance of women in subscales assessing anxiety and sleeping disorders may be related to the fact that the number of older women is bigger than men in many countries in the world, and the level of ensured social security is lower. In the study by Cabak et al. [45], patients with backache showed a much lower level of well-being in terms of mental health than respondents from the control group. Comparable differences were noted in the case of both women and men with chronic pain. However, in this case, men were more prone to depression.

### Occurrence of depressive symptoms

After dementia, depression is the second most frequently occurring psychopathological syndrome in older adults; dementia and depression belong to so-called geriatric giants (next to, e.g., falls, mobility disorders, urinary and fecal incontinence, impaired hearing and seeing) [46]. Compared with the younger population, depression in older adults is characterized by more differentiated and complex etiology and specifics of the clinical presentation, with frequent co-occurrence of somatic symptoms. Depressive disorders in older adults are often chronic and may greatly affect the quality of their health and life. Not only are they the cause of suffering in older adults, impairing their functioning and life quality, they are also accompanied by other diseases, consequently increasing mortality [47–49].

Overall prevalence of depressive symptoms in our study group was around 18%. Results from the literature showed that the prevalence of depression in the world varied from study to study. In China, the prevalence of depression was 3.86% [50]. The incidence of depressive symptoms among older adults in Bangladesh was 45% [51]. The frequency of depressive symptoms in older adults in Turkey was 38.7% [52]. Differences in the sampling of subjects could have resulted in the observed differences in depression prevalence in the elderly in these studies. Compared with the results from the Turkish study [52], including people aged 60+, our prevalence is lower, which could be related to the volunteer group in our study. Summing up, the prevalence and risk of depression in our study were lower than the results of other studies, such as by Jo et al. [53]. Their results differed from the results of studies conducted on younger adults in Korea [54]. A meta-analysis of studies of people over the age of 75 indicated that the incidence of major depression to be 7.2%. Values for women ranged from 4.0% to 10.3% and for men from 2.8% to 6.9% [55]. The frequency of major depression is estimated to be between 10% and 20% in the general older adults [56] and 5% and 17% in primary care conditions [57]. The prevalence of depressive symptoms is reportedly higher than that of depression [58]. In Poland, few studies have been conducted to assess the incidence of depression in the elderly. In the WOBASZ study, the symptoms of depression were found in more than 25% of the examined population [59]. The PolSenior study reported that the morbidity of depressive disorders increased with age (20% in the 55–59 age group, 25% in the 65–79 age group, 33% in those 80 and over) [1].

In our study, the overall BDI result showed that the feeling of the severity of depressive symptoms may have indicated mild depressive disorders. In the study by Goldberg et al. [60] depressive symptoms were highly prevalent and they indicated major depressive disorders. In our study, sex was not significantly related to BDI point values. However, from the statistical point of view, group affiliation and age significantly affected the BDI results. Similarly to our study, the results in the study by Segal et al. [61] suggested no statistically significant relationship between the influence of age and sex on the BDI results.

Right after dementia, depression is the main factor causing increased health care costs for older adults. It is predicted that by 2020 it will be the main cause of morbidity among seniors [62, 63].

### Limitations of the study

The main limitation of the study was the small size of the study groups. In addition, the study was conducted only in one city (Bialystok), which may not reflect the results for the whole country. The study involved only residents of Bialystok, as the authors are employees of the Medical University of Bialystok; therefore, it was easier to access the inhabitants of this city. Another limitation was the selection of a test group. There was a risk that the retirement university communities may have been less suitable for comparison with nursing home residents. They were probably not the best choice for true representation of the whole population of the region, as they were likely to have better physical health and be more prosperous than other members of the community. Moreover, they may have been more affluent than inhabitants of nursing homes. In the analysis, only the raw prevalent data were presented without adjustments for physical limitations, chronic diseases, etc. Differences in mental distress amongst older adults and those in nursing homes were likely due to the presence of other factors, e.g. pain affecting the participants’ mental state, rather than due to being old or living in a nursing home.

## Conclusions

Subjective assessment of mental health status in older adults, inhabitants of Bialystok, was negative. Social and demographic characteristics (sex, group affiliation, age) analyzed in the study played no significant role in the assessment of depressive and non-psychotic mental symptom occurrence. Residents of the nursing home were characterized negatively in terms of subjective assessment mental health status from the other two study groups.

## Abbreviations

GHQ-28: General Health Questionnaire-28
BDI: Beck Depression Inventory
WHO: World Health Organization
UHS: University of Healthy Senior
UTA: University of the Third Age
NH: Nursing Home
